# Effect of Sodium Sulfate on Fracture Properties and Microstructure of High-Volume Slag-Cement Mortar

**DOI:** 10.3390/ma19010043

**Published:** 2025-12-22

**Authors:** Ruizhe Si, Xiangyu Han, Yue Zhang, Haonan Zeng

**Affiliations:** 1 School of Civil and Architectural Engineering, Southwest University of Science and Technology, Mianyang 621000, China; 2 Institute of Civil Engineering Materials, Southwest Jiaotong University, Chengdu 610031, China; 3 State Key Laboratory of Bridge Safety and Resilience, China Merchants Chongqing Communications Research & Design Institute Co., Ltd., Chongqing 400067, China

**Keywords:** fracture properties, mechanical properties, high-volume slag mortar, sodium sulfate, microstructure

## Abstract

This study investigates the effect of added sodium sulfate on the performance of high-volume slag-cement mortar (HVSCM). Herein, Na_2_SO_4_ (0, 1, 2, and 4 wt.% Na_2_O) was used to modify HVSCM. The compressive strength, fracture properties, microstructure, and environmental impact of the synthesized samples were analyzed. The results showed that the 1 day compressive strength of HVSCM can be improved by 345.5% with the addition of 4% Na_2_O (as Na_2_SO_4_), compared to samples without Na_2_SO_4_. However, the 28 day compressive strength of Na_2_SO_4_-activated HVSCM was 14.3–26.4% lower than that of the non-activated HVSCM, though still comparable to OPC. Regarding fracture properties, the initial fracture toughness of non-activated HVSCM was 45.6% higher than that of Ordinary Portland cement (OPC) mortar. Furthermore, Na_2_SO_4_ activation further increased initial fracture toughness, with the sample containing 4% Na_2_O showing a 101.1% improvement over OPC. In contrast, fracture energy was not significantly influenced by Na_2_SO_4_ addition. Microstructurally, the enhanced fracture properties of non-activated HVSCM were attributed to a higher degree of C-(A)-S-H polymerization and a denser binder phase. Sodium sulfate introduced sodium ions to strengthen electrostatic attraction and cohesion between C-(A)-S-H globules, offsetting reduced polymerization. Environmental assessment confirms that both activated and non-activated HVSCM substantially reduce embodied energy and CO_2_ relative to OPC, while the additional embodied energy associated with Na_2_SO_4_ activation remains limited (<12%). Overall, this work provides a comprehensive understanding of the fracture behavior of Na_2_SO_4_-activated HVSCM, elucidating its capacity to enhance early-age strength and fracture toughness while highlighting its limited effect on long-term strength and fracture energy. These findings support the tailored use of Na_2_SO_4_ activation for sustainable construction applications.

## 1. Introduction

Ordinary Portland cement (OPC) is a critical constituent of concrete. Nevertheless, OPC manufacturing is closely associated with severe carbon emissions issues: approximately 0.86 tons of CO_2_ are released per ton of OPC produced [1]. Specifically, the cement industry is responsible for contributing about 8% of anthropogenic CO_2_ emissions [2]. Replacing OPC with supplementary cementitious materials (SCM) in construction materials can reduce the use of the OPC and decrease CO_2_ emissions by around 40% [3]. In addition, cement-SCM blended cementitious binders (such as cement-slag blends and cement-fly ash blends) are reported to exhibit similar or even superior mechanical properties and durability to those of pure OPC systems at later ages [4,5,6]. Thus, the use of eco-friendly cement-SCM blends for construction is an achievable approach to alleviate the adverse effects of cement production on the environment [7].

Ground granulated blast-furnace slag (GGBS) is a type of industrial waste widely used as an SCM to partially replace the OPC in concrete for performance modification [8]. The CO_2_ emissions associated with the production of GGBS are less than 20% of the CO_2_ emissions from the production of the OPC [9,10], highlighting its low-carbon advantage. Partial substitution of OPC with GGBS in cementitious materials enhances long-term strength [5], reduces permeability [11], enhances corrosion resistance [12], and boosts the durability of the binding materials [13]. However, due to the relatively low reactivity of the slag compared to OPC, cementitious materials with high-volume slag (replacement level of slag higher than 50%) exhibit low early-age strength, limiting their practical application [14]. To address the insufficient early-age mechanical performance of high-volume slag cementitious materials, various methods such as high-temperature curing, the use of finer slag particles, and chemical activation have been adopted [5]. Based on the existing research, chemical activation is widely regarded as the most viable method for enhancing the early-age strength of high-volume slag cementitious materials [15].

While commonly used chemical activators (such as NaOH, Na_2_SiO_3_ solutions) have been extensively investigated for early-age performance improvement of high-volume slag-cement mortar (HVSCM), their high embodied energy, safety concerns, and high cost hinder large-scale adoption. In contrast, the use of Na_2_SO_4_ solution as an activator offers a greener alternative due to its low production energy, cost-effectiveness, and potential utilization of industrial by-products [16,17]. Na_2_SO_4_ addition significantly boosts the compressive strength of the cement-slag blends at early ages by increasing the pH value in the system [5]. However, the development of the late-age compressive strength of the cement-slag blends tends to be limited by the added Na_2_SO_4_ [7]. Thus, Na_2_SO_4_ addition exerts a direct regulatory effect on the compressive strength development of cement-slag blends.

Beyond compressive strength, fracture properties are of pivotal importance to cementitious materials. Owing to the intrinsic brittleness of cementitious materials, concrete structures are highly susceptible to external loads, which readily induce crack initiation and propagation, ultimately impairing both structural performance and long-term durability [18]. For high-volume slag cementitious materials, a systematic investigation into their fracture properties is therefore critical for guiding material design and reliable performance assessment.

However, relevant research in this field remains limited, particularly regarding two core aspects: fracture properties of high-volume slag cementitious materials and the influence of Na_2_SO_4_ activation on the fracture behavior of slag-cement blends. Zhu et al. [19] reported that the fracture energy and toughness of engineered cementitious composites (ECC) tended to increase when the slag content increased from 50% to 80% in the mixtures. Özbay et al. [20] found that increasing the level of slag substitution in ECC by up to 80% can lead to a reduction in residual crack width and an enhancement of ductility. The existing studies confirmed that the addition of slag can potentially improve the fracture properties of cementitious materials. Despite progress in understanding compressive strength, a critical gap exists in knowledge regarding the influence of Na_2_SO_4_ on the fracture behavior of HVSCM. This gap hinders a comprehensive understanding of the Na_2_SO_4_-activated HVSCM overall performance and restricts its safe and effective engineering application.

To comprehensively study the fracture behavior of slag-cement blends activated by Na_2_SO_4_, compressive strength and three-point bending tests were performed in this study to assess the mechanical and fracture characteristics of the Na_2_SO_4_-activated slag-cement blends. Fourier-transform infrared spectroscopy (FTIR) analysis was used to investigate the evolution of key reaction products, while X-ray diffraction (XRD) tests were performed to reveal the phase compositions of slag-cement blends activated by Na_2_SO_4_. Scanning electron microscopy with energy-dispersive X-ray spectroscopy (SEM-EDS) tests were conducted to elucidate the microstructure of the samples. The environmental impacts of the prepared cementitious mortars were analyzed in this study. The study contributes to the informed design of sustainable Na_2_SO_4_-activated slag-cement blends, promoting the practical application of these low-carbon cementitious materials.

## 2. Materials and Methods

### 2.1. Materials

**Figure 1:**
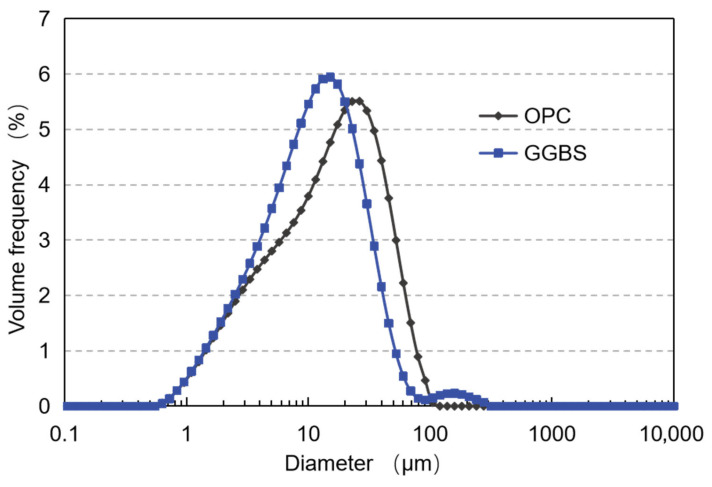
Particle size distribution of raw materials.

The PO 52.5 ordinary Portland cement and S95 GGBS from Zhucheng Jiuqi Building Materials Co., Ltd., Weifang, China were used in this study as cementitious materials. The chemical compositions of the OPC and GGBS measured by the X-ray fluorescence (XRF) test are shown in Table 1. Figure 1 illustrates the particle size distribution of the OPC and GGBS. The median particle sizes (D50) of cement and GGBS are 14.34 µm and 10.47 µm, respectively. The anhydrous sodium sulfate was used for the preparation of the activators in this study. Well-graded sand was selected as the fine aggregate for sample preparation.

### 2.2. Mixture Design and Preparation Process

The mortar specimens were prepared with a 3:7 cement-to-slag ratio and activated with Na_2_SO_4_ solutions containing 0, 1, 2, and 4 wt.% of Na_2_O relative to the total binder mass. A group of mortar samples prepared with OPC was used as the control group. A consistent water-to-binder ratio (0.45) and sand-to-binder ratio (2.25) were adopted for all mortar mixtures. The detailed mix proportions of the designed samples are presented in Table 2. The mix ID in Table 2 indicates the type of the prepared samples, ‘OPC’ indicates cement mortar without slag addition, ‘S’ and ‘N’ indicates the content of slag in the binder of the mortar sample and Na_2_O concentration in the activator, respectively (e.g., S70N1 indicates the mortar samples prepared with 70% of slag for the binder and 1 wt.% of Na_2_O for the activator).

Twenty-four hours before mixing, Na_2_SO_4_ solutions with different Na_2_O contents were prepared. The following protocol was used for mortar preparation: first, dry ingredients (cement and slag) were combined in a mixer for 2 min. Then, within 30 s, water or an activator was added. Next, sand was introduced, and the mix was stirred for 3 min at low speed, followed by high-speed stirring for 2 min. Finally, the mortar was immediately cast into molds and vibrated for 30 s to ensure proper compaction and eliminate air bubbles.

### 2.3. Methods

#### 2.3.1. Compressive Strength

According to ASTM C109 standard [21], compressive strength was evaluated using 50 mm × 50 mm × 50 mm cube specimens subjected to a constant loading rate of 1 kN/s. Tests were performed at 1, 2, 3, 7, and 28 days to monitor the development of compressive strength. To ensure statistically robust results, three replicates were tested for each mix composition.

#### 2.3.2. Fracture Properties

**Figure 2:**
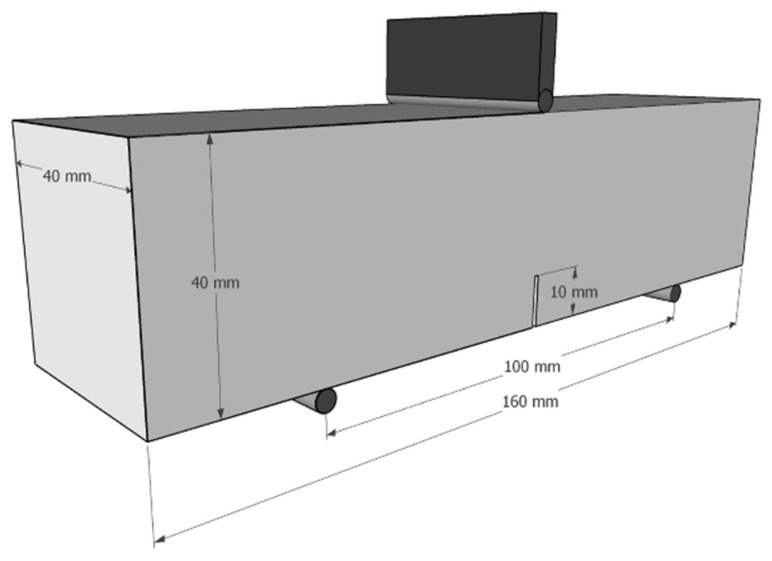
Dimensions of the TPB test mortars.

The fracture properties of OPC and Na_2_SO_4_-activated slag-cement mortars were determined by using three-point bending (TPB) tests at 28 days. Beam samples with dimensions of 40 mm × 40 mm × 160 mm were prepared, each containing a single 10 mm edge notch, as presented in Figure 2. The TPB test utilized a 100 mm center-to-center support span.

The prepared samples were sealed with plastic film and cured at ambient temperatures after demolding until the age of 28 days. The initial fracture toughness ($K_{iC}^{I}$) and unstable fracture toughness ($K_{iC}^{U}$) were used to assess the fracture toughness of the OPC and slag-cement blended mortars [22,23]. A clip gauge was used to collect the crack mouth opening displacement (CMOD) of the specimens. Equations (1) and (2) were employed to calculate the $K_{iC}^{I}$ [24]:

(1) $$K_{iC}^{I}=\frac{3P_{ini}S\sqrt{a_{0}}}{2H^{2}W}f\left(\alpha_{0}\right)$$

(2) $$f\left(\alpha_{0}\right)=\frac{1.99-\alpha_{0}(1-\alpha_{0})(2.15-3.93\alpha_{0}+2.7\alpha_{0}^{2})}{(1+2\alpha_{0})(1-\alpha_{0}{)}^{\frac{3}{2}}}$$

where $a_{0}$ is the notch depth of 10 mm; S, H, and W are the span (100 mm), height (40 mm), and width (40 mm) of the sample; $\alpha_{0}$ = $a_{0}$/H, and $P_{ini}$ denotes the load causing the initial cracking. $P_{ini}$, corresponding to the beginning of the non-linear segment, was carefully determined by analyzing the load-CMOD curves [25]. Equations (3) and (4) were used to determine the $K_{iC}^{U}$ [24]:

(3) $$K_{iC}^{U}=\frac{3P_{max}S\sqrt{a_{c}}}{2H^{2}W}f\left(\alpha_{c}\right)$$

(4) $$f\left(\alpha_{c}\right)=\frac{1.99-\alpha_{c}(1-\alpha_{c})(2.15-3.93\alpha_{c}+2.7\alpha_{c}^{2})}{(1+2\alpha_{c})(1-\alpha_{c}{)}^{\frac{3}{2}}}$$

where $\alpha_{c}$ = $a_{c}$/H, the critical effective length ($a_{c}$) was obtained by Equations (5) and (6) [24]:

(5) $$a_{c}=\frac{2H}{\pi }arctan{\left(\frac{E\times W\times {CMOD}_{e}}{32.6P_{max}}\right)}^{\frac{1}{2}}$$

(6) $$E=\frac{1}{Wc_{i}}\left[3.7+32.6tan^{2}\left(\frac{\pi }{2}\frac{a_{0}}{H}\right)\right]$$

where *P_max_* is the maximum load, ${CMOD}_{e}$ is the value of CMOD at $P_{max}$, E represents the modulus of elasticity of prepared samples, W is the width (40 mm) of the sample, $c_{i}$ denotes the gradient of the linear portion of the load-CMOD curve, and H is the height (40 mm) of the sample. The fracture energy ($G_{f}$) can be calculated by Equation (7) [24]:

(7) $$G_{f}=\frac{W_{0}+mg\delta_{0}}{A_{lig}}$$

where *W*_0_ denotes the external work, which can be obtained by integrating the load-deflection curve during the TPB test; *δ*_0_ denotes the mid-span displacement; m is the mass of the sample; g is the gravity constant (9.8 m/s^2^); A_lig_ represents the area of the fractured ligament calculated by specimen dimensions.

#### 2.3.3. XRD

The OPC binder and slag-cement blended binder with varying Na_2_SO_4_ contents (without fine aggregate) were used for the XRD test after curing for 28 days under sealed conditions and ambient temperature. The XRD measurements were conducted using Cu Kα radiation (45 kV, 40 mA), scanning from 5° to 65° 2θ (0.026 2θ step size).

#### 2.3.4. FTIR

The Nicolet iS50 was utilized for the FTIR measurements. The tested samples were prepared by mixing 1 mg of the paste samples (without fine aggregate) with 10 mg of KBr to form a homogeneous pellet. FTIR spectra were acquired with a resolution of 2 cm^−1^ in the mid-infrared range (4000–400 cm^−1^). FTIR spectra within the range from 1300 to 750 cm^−1^ were deconvoluted by employing Gaussian peak shapes and considering the positions of various functional peaks.

#### 2.3.5. SEM-EDS

Microstructural analysis of fractured mortar samples obtained from 28 day TPB tests was conducted by SEM. The EDS analysis was conducted on the paste samples; the selected points for the tests were within the binder area, avoiding the unreacted raw materials [26].

#### 2.3.6. Environmental Impact Analysis

The environmental impacts of the synthesized OPC and slag-cement blended samples with different Na_2_SO_4_ contents were evaluated in this study. The consumption of energy and carbon dioxide emissions for the production of the prepared mixes in this study were analyzed based on embodied energy (EE) as well as embodied carbon dioxide emissions (*ECO*_2_), respectively [27]. The embodied carbon dioxide index (ECI) and embodied energy index (EEI) were also evaluated in this study [28,29]. The ECI and EEI can be calculated by Equations (8) and (9), respectively [29].

(8) $$ECI=\frac{embodied\;CO_{2}(kg/ton)}{\sigma (MPa)}$$

(9) $$EEI=\frac{embodied\;energy(MJ/ton)}{\sigma (MPa)}$$

where σ refers to the 28 day compressive strength of mortars.

## 3. Results

### 3.1. Compressive Strength

**Figure 3:**
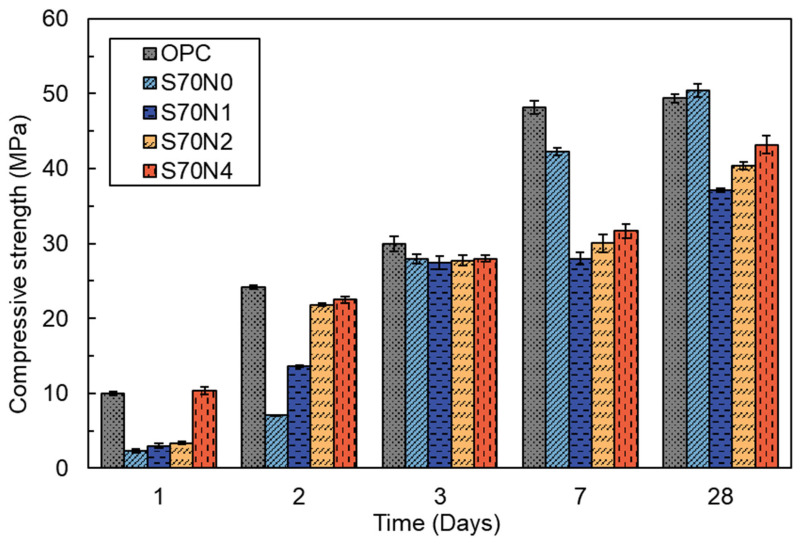
Compressive strength of the cement-slag blended samples activated with different concentrations of Na_2_SO_4_ solution.

Figure 3 illustrates the compressive strength results of OPC mortar and slag-cement blended mortar activated with varying Na_2_SO_4_ contents. Compared to the OPC sample, the S70N0 sample exhibited lower compressive strength within the first 7 days. However, the compressive strength of S70N0 achieved a slightly higher 28 day compressive strength due to the pozzolanic reaction of the slag [30]. Na_2_SO_4_ activation resulted in a significant increase in the early-age (within 2 days) compressive strength of slag-cement blends, demonstrating a direct correlation between concentration and early-age strength improvement. The 1 day compressive strength of the S70N1, S70N2, and S70N4 was increased by 27.53%, 42.71%, and 345.54%, respectively, compared to the S70N0. Particularly, the compressive strength of S70N4 was comparable to that of the OPC at the age of 1 day. Increasing Na_2_SO_4_ content from 1% to 4% in slag-cement blended mortar boosted the compressive strength from 37.1 MPa to 43.2 MPa at 28 days. However, the compressive strength of S70N1, S70N2, and S70N4 reduced by 26.4%, 20.0%, and 14.3%, respectively, compared to that of S70N0 at 28 days. The reasons driving this strength change due to high slag volume and Na_2_SO_4_ addition are discussed in Section 4.

### 3.2. Fracture Properties

**Figure 4:**
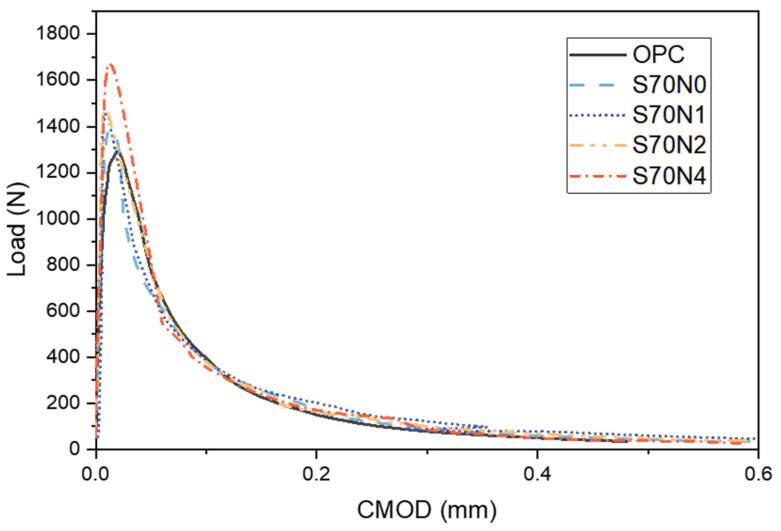
Load-CMOD curves of the prepared OPC mortar and slag-cement blended samples.

Representative Load-CMOD curves, reflecting the bending response, for the prepared OPC and Na_2_SO_4_-activated or non-activated slag-cement blended mortar samples are depicted in Figure 4. The curves exhibit similar characteristics for all samples, which can be categorized into three stages: an initial linear increase stage, a crack propagation stage, and a failure stage. This indicates that the prepared OPC and slag-cement blended mortar samples can be considered quasi-brittle materials. The peak load of the slag-cement blended mortar samples was generally higher than that of OPC. Particularly, the enhancement of the Na_2_SO_4_ concentration promoted the improvement of the peak loads of the slag-cement blends, and the S70N4 sample achieved the highest peak load among the tested mortars.

#### 3.2.1. Fracture Toughness

**Figure 5:**
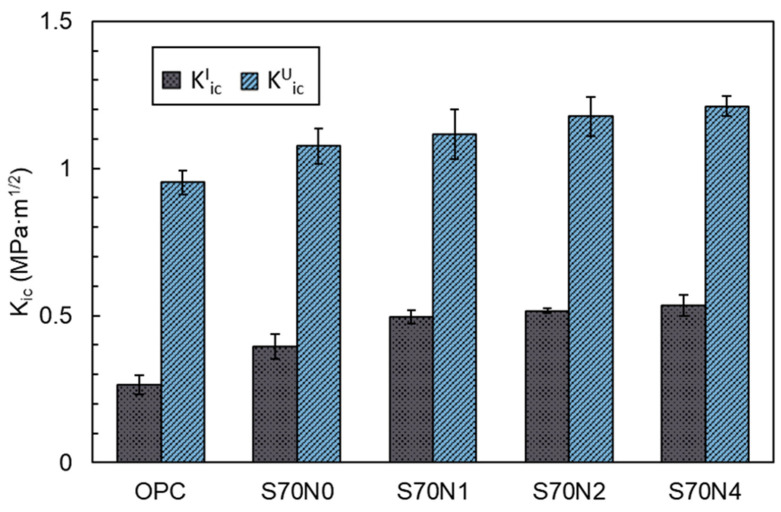
Fracture toughness of OPC and high-volume slag mortar samples with different contents of Na_2_SO_4_.

The $K_{iC}^{I}$ and $K_{iC}^{U}$ of the OPC mortar and high-volume slag mortar samples with or without Na_2_SO_4_ activation are shown in Figure 5. The $K_{iC}^{I}$ of the S70N0 sample increased by 45.6% compared to the OPC sample, indicating that the incorporation of a high volume of slag improved the resistance to crack initiation of the cement mixture. Additionally, the $K_{iC}^{I}$ of the high-volume slag mortar was further enhanced with an increase in the Na_2_SO_4_ concentration. Among the prepared mortar samples, the S70N4 sample achieved the highest $K_{iC}^{I}$, which increased by 101.1% and 35.3% relative to the OPC and S70N0 samples, respectively. This implies that the use of the Na_2_SO_4_ activation method is beneficial for improving the resistance to crack initiation in slag-cement blends.

As shown in Figure 5, the results of the $K_{iC}^{U}$ generally followed the change trend of the $K_{iC}^{I}$. The $K_{iC}^{U}$ of the slag-cement blended mortar can be enhanced by introducing a high volume of slag and the use of Na_2_SO_4_ activation compared to OPC mortar. This increase in the $K_{iC}^{U}$ can be related to the change in the properties of reaction products and microstructure of the mortar, which is further discussed in Section 4.

#### 3.2.2. Fracture Energy

**Figure 6:**
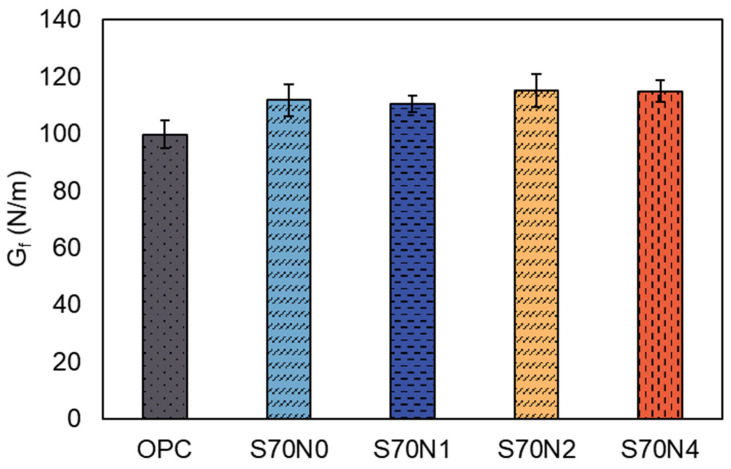
Fracture energy of OPC and high-volume slag mortar samples with different contents of Na_2_SO_4_.

Fracture energy is one of the crucial parameters to reflect the fracture performance of cementitious materials. Figure 6 presents the fracture energy of the prepared OPC and slag-cement blended mortar samples. It can be seen that the fracture energy of the S70N0 is enhanced by 12.04% compared to that of OPC. Yazıcı et al. [31] reported similar findings, observing an improvement in fracture energy when incorporating high-volume slag into blended systems. They attributed this enhancement to the improved bonding strength resulting from slag incorporation. In addition, the impact of Na_2_SO_4_ content on the fracture energy of the mortar was negligible.

As shown in Figure 6, the non-activated slag mortar (S70N0) achieved a $G_{f}$ of 111.7 N/m. With Na_2_SO_4_ activation, the values ranged from 110.4 N/m (S70N1) to 115.1 N/m (S70N2). The $G_{f}$ of the sample with the highest activator dosage (S70N4, 114.8 N/m) was only 2.7% higher than that of S70N0. This confirms that the influence of Na_2_SO_4_ content on fractured energy was statistically marginal. This observation presents a noteworthy contrast to the significant enhancement in fracture toughness ($K_{iC}^{I}$ and $K_{iC}^{U}$) caused by Na_2_SO_4_ activation (Figure 5). While the activator markedly improved the resistance to crack initiation and propagation (toughness), it did not substantially alter the total energy absorption capacity ($G_{f}$) of the high-volume slag matrix. This indicates that the mechanisms governing these two fracture parameters are distinct: fracture toughness is highly sensitive to the local cohesion at the crack tip, which is strengthened by Na^+^ ions, whereas fracture energy is governed by the overall microstructural integrity and crack path tortuosity, which are predominantly conferred by the high-volume slag content itself. This point is elaborated further in Section 4.

### 3.3. XRD Analysis

**Figure 7:**
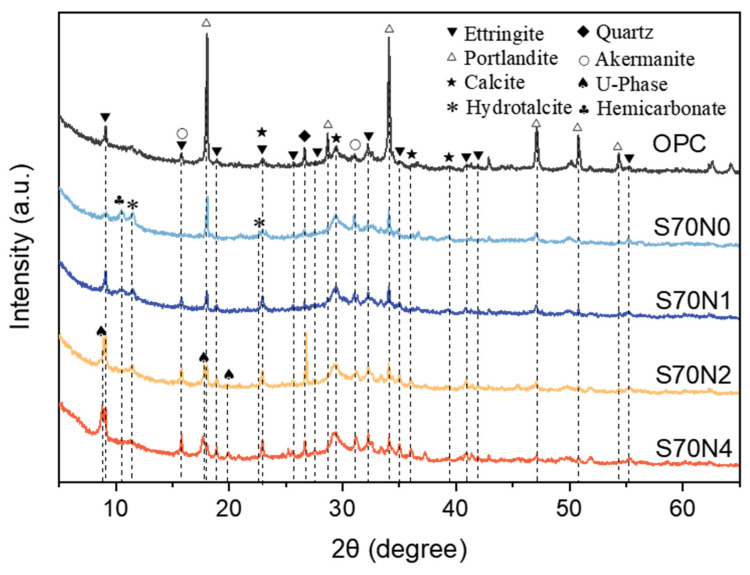
XRD patterns of OPC and slag-cement mortar with different content of Na_2_SO_4_.

The XRD spectra of all prepared samples at 28 days are shown in Figure 7. The crystal phases, including portlandite, quartz, calcite, and akermanite, can be observed in all prepared samples according to the XRD results illustrated in Figure 7. The presence of quartz in the samples was due to impurities in the raw materials. The formation of calcite could be caused by the natural carbonation of the samples by atmospheric CO_2_ during the sample preparation and curing [32]. The peak intensity of the portlandite was lower in samples with a high volume of slag compared to the OPC sample. This is due to the higher CaO content in the OPC sample than that in the mixtures with high-volume slag, leading to the formation of more portlandite in the mixture [33]. Additionally, the portlandite formed during the hydration of the cement can be consumed by the introduced slag by the pozzolanic reaction, which contributed to the decrease in the portlandite content and enhancement of the calcium (aluminate) silicate hydrates (C-(A)-S-H) in the mixtures with a high-volume slag [34].

A prominent broad peak centered at 29° 2θ is observed in the diffraction patterns of all analyzed samples, corresponding to the formed C-(A)-S-H, the dominant reaction product in cementitious systems [35,36]. The ettringite phase can be identified in all prepared samples, and the intensity of the ettringite increased with the Na_2_SO_4_ content for slag-cement mixtures.

Hydrotalcite (Ht) and Hemicarbonate (Hc) can be identified as the secondary phases in cementitious materials with a high content of slag [37,38,39]. The formation of the Hc can be observed in S70N0 and S70N1 samples; however, the Hc peak was not found when the Na_2_SO_4_ content was higher than 2% in the mixtures. The peak intensity of the Ht in the samples with high-volume slag was decreased as the Na_2_SO_4_ content was enhanced. The U-phase was shown in the S70N2 and S70N4 samples, which was attributed to the high concentration of Na_2_SO_4_ in the mixtures [40].

### 3.4. SEM and EDS Analysis

**Figure 8:**
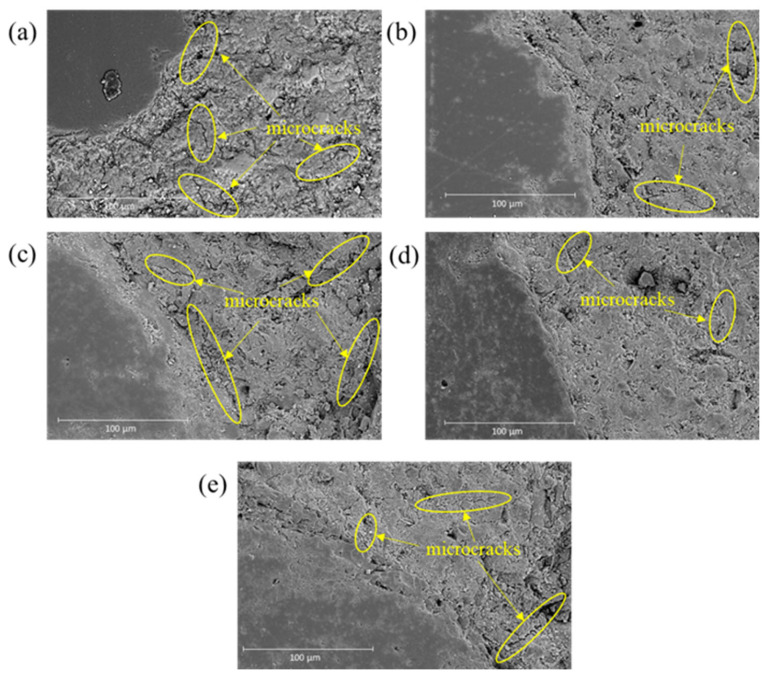
SEM images of the prepared OPC and slag-cement mortar. (**a**) OPC; (**b**) S70N0; (**c**) S70N1; (**d**) S70N2; (**e**) S70N4.

Figure 8 presents SEM images of the microstructure of OPC and slag-cement blended mortar samples following the TPB test. The matrix of slag-cement blended mortar was denser than that of OPC mortar. In addition, the interfacial transition zone (ITZ) of the slag-cement blended mortar with different Na_2_SO_4_ tended to be denser than that of the OPC mortar. This phenomenon was in line with the study of Ding et al. [41]. They found that the slag-based cementitious materials presented a more homogenous matrix and stronger ITZs than ordinary Portland cement mortar. As shown in Figure 8, the microcracks were primarily generated at ITZ areas and within the matrix part of the samples. This indicates that the fracture of the prepared mortars generally occurred within the matrix and ITZ area. Therefore, the fracture properties of the prepared mortars were mainly determined by the performance of the ITZ and binder phase of the mortar.

**Figure 9:**
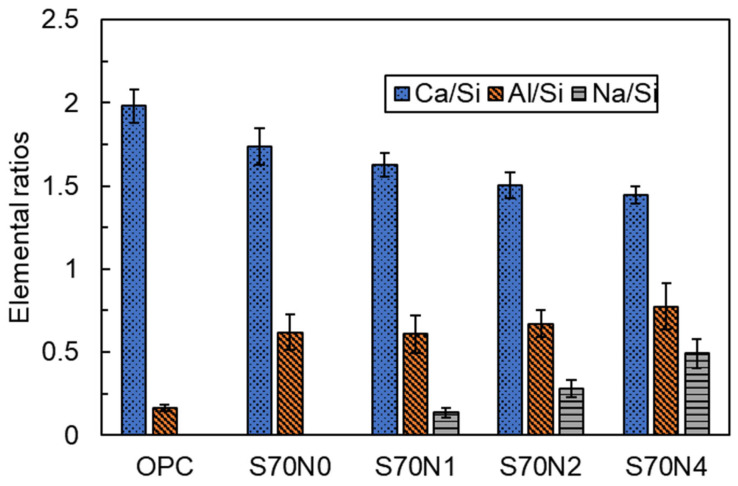
Mean Ca/Si, Al/Si, and Na/Si atom ratios obtained by EDS test for OPC and slag-cement blends with/without Na_2_SO_4_.

EDS analysis revealed the chemical composition of reaction products of the OPC and slag-cement blended pastes with and without Na_2_SO_4_ addition. C-(A)-S-H gel, as previously reported, was identified as the primary reaction product in both OPC and blended samples [42]. Figure 9 presents the estimated Al/Si, Na/Si, and Ca/Si ratios of the C-(A)-S-H gel within the prepared mixtures, obtained from EDS data. Substituting 70 wt.% cement with slag in the mixture significantly reduced the Ca/Si ratio of the C-(A)-S-H gel, dropping from 1.98 to 1.73. Conversely, the Al/Si ratio experienced a substantial increase, rising from 0.16 to 0.65, compared to the OPC mixture. This phenomenon is due to the introduced slag possessing a relatively low content of Ca and a high content of Al compared to traditional cement. The observed alterations in the Al/Si and Ca/Si ratios of C-(A)-S-H align with analogous trends identified in other blended cement mixtures with aluminosilicate-rich SCMs [43]. For Na_2_SO_4_-activated slag-cement blends, the Al/Si and Na/Si ratios of the samples increased with increasing Na_2_SO_4_ content. This is due to the higher content of introduced Na_2_SO_4_, which enhanced the concentration of Na^+^ in the mixture, and the Na^+^ ions tended to improve the incorporation of Al into the C-(A)-S-H gel [44]. The Ca/Si ratio of the Na_2_SO_4_-activated slag-cement blends decreased with the enhanced Na_2_SO_4_ concentration and increased Na/Si ratio in the mixtures. The reduction in the Ca/Si ratio in C-S-H with the introduction of alkali content was also reported by Lodeiro et al. [45].

### 3.5. FTIR Analysis

**Figure 10:**
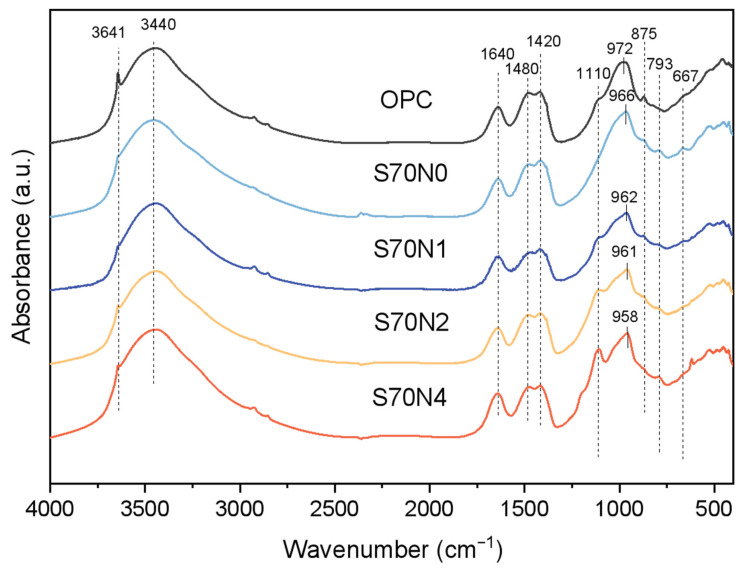
FTIR spectra of OPC and slag-cement blended paste with various Na_2_SO_4_ contents.

FTIR analysis (Figure 10) provided insights into the reaction products of OPC and slag-cement blended mixtures. The peak at 3641 cm^−1^ corresponds to hydroxyl groups (OH) associated with calcium hydroxide [46]. Its intensity decreased with slag and Na_2_SO_4_ incorporation, suggesting reduced Ca(OH)_2_ content in the mixture. Bands around 3440 cm^−1^ as well as 1640 cm^−1^ indicate water molecules within the reaction products, while peaks at 1480 cm^−1^ [47], 1420 cm^−1^ [48], and 870 cm^−1^ [49] confirmed carbonation of the samples. Additionally, the 1110 cm^−1^ band indicates S-O vibrations corresponding to ettringite formation [50]. These FTIR findings align with the XRD results.

FTIR analysis (Figure 10) reveals changes in the amorphous C-(A)-S-H structure within OPC and slag-cement blends, focusing on the broad Si–O–T (T represents Si or Al) vibration bands (950–1000 cm^−1^) [46,51]. The primary peak position shifts from 972 cm^−1^ in OPC to 966 cm^−1^ in S70N0, indicating potential structural modifications. This shift could be attributed to two factors: (i) an increased replacement ratio of the Si by Al in C-(A)-S-H gel [44]. Consistent with EDS data (Figure 9), S70N0 shows a significantly higher Al/Si ratio compared to OPC, supporting this explanation; (ii) a reduction in the polymerization degree of C-(A)-S-H [52]. While some studies [53] suggest a higher Ca/Si and Al/Si ratio can enhance polymerization, leading to a higher Si–O–T wavenumber, this does not appear to be the case in this study. Therefore, the observed shift in the Si–O–T position likely arises primarily from the enhanced Al incorporation in C-(A)-S-H of S70N0, with the polymerization degree playing a lesser role in this situation.

The Si–O–T wavenumber value decreased from 966 cm^−1^ to 958 cm^−1^ as the Na_2_SO_4_ content increased from 0 to 4% in slag-cement blended mixtures. In addition to the increase in Al/Si ratios with enhanced Na_2_SO_4_ content, the incorporation of alkali ions (Na^+^) into the C-(A)-S-H might be one of the reasons for the shift in wavenumber observed in slag-cement blended mixtures with varying Na_2_SO_4_ content [54]. The existing literature suggests that the incorporation of alkali cations can shorten mean chain lengths and decrease the degree of silicate polymerization in C-(A)-S-H gel [55], which could result in the shift in the wavenumber of the samples to the lower position [52].

**Figure 11:**
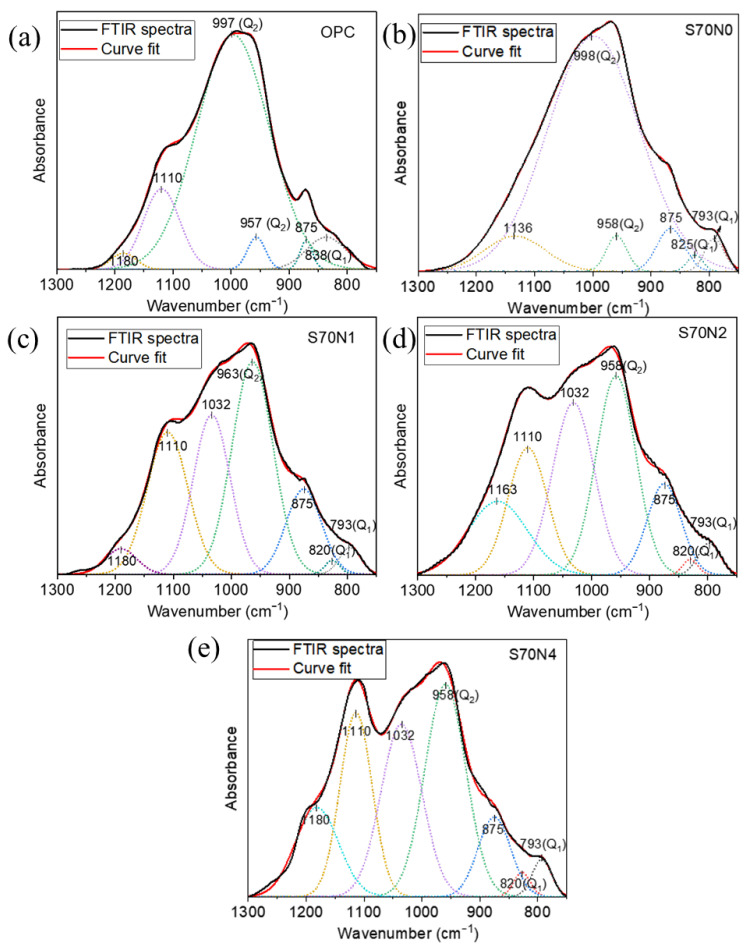
Deconvolution of FTIR spectra of the synthesized samples. (**a**) OPC; (**b**) S70N0; (**c**) S70N1; (**d**) S70N2; (**e**) S70N4.

The wavenumber ranging from 1300 to 750 cm^−1^ associating Si–O or Al–O bonds in prepared samples was deconvoluted to analyze the change in C-(A)-S-H structure in prepared samples [56], as shown in Figure 11. The peak at around 1032 cm^−1^ is associated with the silicate-based gel attached with Na^+^ ion [57,58]. The bands located within the range from 1135 to 1180 cm^−1^ are Si–O bonds in quartz [59,60,61]. The information of the Si-O tetrahedra of Q_2_ units can be indicated by the peak from 950 to 1000 cm^−1^ [62], while the Si–O stretching vibration of Q_1_ units is indicated by the peaks between 790 and 840 cm^−1^ [62,63], as shown in Figure 11. The ratio of Q_2_ to Q_1_ area obtained from deconvoluted results could reflect the polymerization degree of the C-(A)-S-H. The results of the relative area of Q_2_ units and Q_1_ units, as well as their ratio, are summarized in Table 3. The S70N0 has a higher ratio of Q_2_ peak to Q_1_ peak area than that of OPC, which indicates the enhanced polymerization degree of C-(A)-S-H in S70N0 samples. This can be due to the introduced Al species, enhanced polymerization of the aluminosilicate chains in the C-(A)-S-H structure [64]. This result is in line with the results of EDS tests, as the Al/Si ratio increased in the S70N0 compared to that in OPC. The increase in Na_2_SO_4_ content from 0 to 4% in the slag-cement blended mixtures results in a reduction in the ratio of Q_2_ peak to Q_1_ peak area of the tested samples, as shown in Table 3. This confirmed that the added Na_2_SO_4_ introduced alkali ions and reduced the mean chain lengths and degree of polymerization of C-(A)-S-H gel [55].

### 3.6. Environmental Impact Analysis

**Figure 12:**
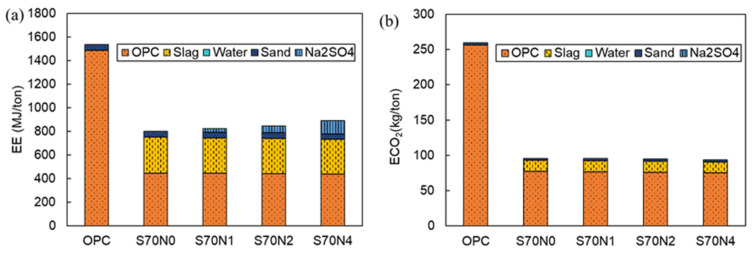
Proportion of embodied energy and embodied carbon dioxide emissions of each raw material for the prepared mortar. (**a**) EE; (**b**) ECO_2_.

The EE and ECO_2_ of the raw materials for the OPC and slag-cement blended mortars with varying Na_2_SO_4_ contents prepared in this study are shown in Table 4, which were employed to calculate the sustainability parameters of the cementitious mortar mixtures in this study. Particularly, Na_2_SO_4_ can be obtained as an industrial byproduct, and its CO_2_ emissions during manufacturing were often disregarded [17,65]. Figure 12 reveals that cement is the primary contributor to EE and ECO_2_ for OPC mortar. For slag-cement blended mortars, both cement and slag contribute significantly to EE and ECO_2_. Compared to OPC mortar samples, the EE and ECO_2_ of slag-cement blended mortar with varying Na_2_SO_4_ contents were reduced by at least 42.1% and 63.1%, respectively. This implies a substantial reduction in the negative environmental impact of slag-cement blended mortar with or without Na_2_SO_4_ compared to OPC mortar.

Figure 12a shows that adding Na_2_SO_4_ to the slag-cement mixes increases the absolute EE of the prepared mortars. The increase in EE relative to S70N0 remains modest (≤12% for the highest Na_2_SO_4_ dosage tested). By contrast, ECO_2_ is essentially unchanged (Figure 12b) because the Na_2_SO_4_ inventory used here assumes negligible manufacturing CO_2_ (Table 4).

**Figure 13:**
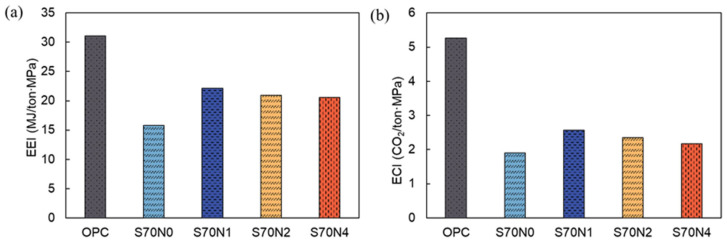
Sustainability analysis of the prepared OPC and slag-cement blended mortar with or without Na_2_SO_4_. (**a**) EEI; (**b**) ECI.

The calculated EEI and ECI of the prepared mortar samples followed a similar trend, as shown in Figure 13. Slag-cement blended mortars with varying Na_2_SO_4_ contents consistently exhibited significantly lower EEI and ECI compared to OPC mortar samples. Notably, the S70N0 sample achieved the lowest EEI and ECI among all prepared mixtures. The addition of Na_2_SO_4_ tended to increase both EEI and ECI of the slag-cement blends. Increasing Na_2_SO_4_ mitigates the increase in EEI and ECI caused by Na_2_SO_4_ itself. The environmental impact analysis confirmed that slag-cement blended mortar with varying Na_2_SO_4_ contents improves the sustainability of cementitious mortars.

## 4. Discussion

The experimental results illustrated the influence of Na_2_SO_4_ activation on the performance of high-volume slag-cement mortar (HVSCM), with distinct effects on early-age versus later-age properties, as well as on different fracture parameters.

The lower early-age compressive strength of the non-activated HVSCM (S70N0) compared to OPC is attributed to the inherently lower reactivity of slag relative to cement clinker [67]. However, by 28 days, S70N0 achieved comparable or even slightly higher strength than OPC. This later-age strength development is facilitated by the pozzolanic reaction of slag, which consumes portlandite and contributes to the formation of additional C-(A)-S-H, resulting in a denser binder phase as confirmed by SEM observations (Figure 8) [30].

The addition of Na_2_SO_4_ significantly enhanced the early-age (1–2 day) compressive strength of HVSCM. This acceleration is primarily driven by the introduced sulfate and alkali ions, which elevate the pore solution pH and promote the early dissolution and hydration of both slag and cement phases [5]. However, this activation led to a reduction in the 28 day compressive strength compared to the non-activated S70N0 mix. XRD analysis revealed a decrease in the portlandite peak intensity with the introduction of Na_2_SO_4_ (Figure 7). As CH is a primary product of OPC hydration, its reduced abundance directly indicates lower OPC clinker reaction. This inhibition of later-age cement hydration by Na_2_SO_4_ addition has also been reported by Fu et al. [5] and Mota et al. [68]. Thus, this decline in later-age strength is likely due to the inhibitory effect of alkali ions on the continued hydration of ordinary Portland cement [68]. Particularly, higher Na_2_SO_4_ content led to an increasing trend in later-age strength for the Na_2_SO_4_-activated slag-cement mixtures. This phenomenon can be attributed to the improved hydration of the slag, facilitated by the increased pH value and reduced activity of Ca^2+^ resulting from the elevated Na_2_SO_4_ content [5].

Regarding fracture properties, the incorporation of a high volume of slag itself (S70N0) improved fracture toughness ($K_{iC}^{I}$ and $K_{iC}^{U}$) and fracture energy ($G_{f}$) compared to OPC. Microstructurally, this enhancement is linked to two key factors in S70N0: (i) a more polymerized C-(A)-S-H gel, as indicated by the higher Q^2^/Q^1^ ratio from FTIR deconvolution (Table 3), and (ii) a denser, more homogeneous matrix and interfacial transition zone (ITZ), as observed via SEM (Figure 8). The increased polymerization is associated with the higher Al/Si ratio in the C-(A)-S-H gel of the slag blend (Figure 9), which can strengthen the aluminosilicate chain structure [69,70,71].

**Figure 14:**
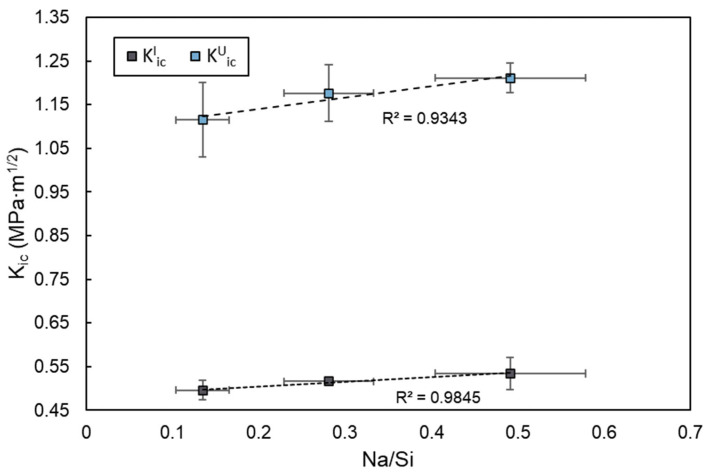
Correlation of fracture toughness as a function of the Na/Si ratio of C-(A)-S-H as the major reaction product of Na_2_SO_4_ activated HVSCM.

The role of Na_2_SO_4_ activation involves a more complex mechanism. It reduces the degree of silicate polymerization in the C-(A)-S-H gel, evidenced by a lower Q^2^/Q^1^ ratio and a shift in the Si–O–T FTIR band to lower wavenumbers (Table 3 and Figure 11). It simultaneously increased both the initial and unstable fracture toughness. This apparent contradiction can be reconciled by considering the nanoscale interaction forces within the binder. The introduced sodium ions are incorporated into the C-(A)-S-H structure, as reflected by the increased Na/Si ratio of C-(A)-S-H (Figure 9). As the C-(A)-S-H globule surfaces are negatively charged, the increased concentration of positively charged Na^+^ cations strengthens the electrostatic attraction and cohesion between adjacent globules [54,72,73]. In addition, the $K_{iC}^{I}$ and $K_{iC}^{U}$ were plotted against the corresponding Na/Si ratio of the reaction product in Na_2_SO_4_-activated blends, as shown in Figure 14. A strong positive linear correlation was observed between the Na/Si ratio and the fracture toughness parameters $K_{iC}^{I}$ and $K_{iC}^{U}$. The correlation indicates that the fractured performance of these blends is positively influenced and largely governed by the sodium ions incorporated into the dominant C-(A)-S-H gel phase. Consequently, the improved resistance to crack initiation and propagation in Na_2_SO_4_-activated blends is attributed not to the change in the polymerization, but to this reinforced cohesion between C-(A)-S-H globules, which effectively offsets the potential weakening effect of a depolymerized silicate network [26,74].

In contrast to fracture toughness, the fracture energy, which represents the total energy dissipated throughout the complete fracture process, encompasses both crack initiation and stable propagation. This parameter is governed more by the overall microstructural integrity, the tortuosity of the crack path, and the bulk homogeneity of the matrix and ITZ [41]. Since these macro-scale, microstructural features are predominantly conferred by the high-volume slag content—resulting in a consistently dense matrix across all slag blends (Figure 8)—and are not substantially altered by Na_2_SO_4_ addition, the $G_{f}$ values remain high and statistically similar regardless of the activator dosage. The minimal variation in fracture energy indicates that the fundamental energy-absorbing capability of the slag-based matrix is preserved, even as the local crack-tip resistance is modulated by the alkali activator.

## 5. Conclusions

This study explored the influence of high-volume slag addition and Na_2_SO_4_ activation on the mechanical performance, fracture performance, and microstructure of cementitious mortar. The main results of the study yield the following conclusions:

1. The compressive strength of the slag-cement blends was lower than that of OPC at early ages but eventually achieved comparable or higher strength by 28 days, owing to the formation of a denser binder phase. The Na_2_SO_4_ activation of HVSCM significantly boosted early-age strength. However, it resulted in a reduction in the 28 day compressive strength compared to the non-activated slag mixture, which is attributed to a decreased later-age hydration degree.
2. Slag-cement blends exhibited higher peak loads and fracture toughness compared to OPC mortars. The generation of more polymerized C-(A)-S-H and denser microstructure contributed to the enhancement of the fracture toughness of the S70N0 samples. The fracture energy of slag-cement blends was also superior to that of OPC, but it remained largely unaffected by the addition of Na_2_SO_4_.
3. Na_2_SO_4_ activation increased Al/Si and Na/Si ratios in C-(A)-S-H gel and promoted the formation of ettringite in the slag-cement blends. The introduced sodium ions reduced the polymerization degree of C-(A)-S-H. Despite this depolymerization, the fracture toughness of Na_2_SO_4_-activated blends increased. This is attributed to the enhanced cohesion between C-(A)-S-H globules, resulting from stronger electrostatic attraction induced by the sodium ions, which improved the resistance to crack initiation and propagation.
4. Environmental analysis showed that both Na_2_SO_4_-activated and non-activated slag-cement blends can significantly reduce embodied energy and CO_2_ emissions compared to OPC, indicating superior sustainability. The addition of Na_2_SO_4_ led to a slight increase in the overall environmental impact.

In summary, Na_2_SO_4_ activation positively influenced the early-age compressive strength, fracture performance, and microstructure of slag-cement blended mortar. The environmental sustainability of slag-cement blends was superior to OPC-based mortars. This study provides crucial insights into the mechanical performance, fracture properties, and environmental sustainability of Na_2_SO_4_-activated slag-cement blends. Building on the observed microstructural changes, future research will include a comprehensive durability assessment of Na_2_SO_4_-activated HVSCM, focusing on long-term sulfate resistance, expansion behavior, and strength evolution under extended curing and aggressive environments to ensure its microstructural stability and long-term engineering performance.

## Figures and Tables

**Table 1:** Chemical compositions of raw materials (wt.%).

	CaO	SiO_2_	Al_2_O_3_	MgO	SO_3_	TiO_2_	Na_2_O	MnO	K_2_O	Fe_2_O_3_	LOI
OPC	52.88	22.71	8.43	4.12	3.85	0.42	0.35	0.14	0.93	3.01	3.16
GGBS	39.74	27.88	17.42	8.39	2.38	1.38	0.41	0.39	0.31	0.3	1.4

**Table 2:** Mix proportions of the prepared mortars.

Mix ID	OPC (%)	Slag (%)	Na_2_SO_4_ (Weight% to Binder)	Water/Binder Ratio	Sand/Binder Ratio
OPC	100	-	-	0.45	2.25
S70N0	30	70	-	0.45	2.25
S70N1	30	70	2.29	0.45	2.25
S70N2	30	70	4.58	0.45	2.25
S70N4	30	70	9.16	0.45	2.25

**Table 3:** Relative area of the deconvoluted T–O components in different samples.

Component	OPC	S70N0	S70N1	S70N2	S70N4
Relative area of Q_1_	5.61	3.36	3.07	3.29	3.89
Relative area of Q_2_	78.00	84.14	34.82	27.71	29.63
Q_2_/Q_1_ area ratio	13.90	25.07	11.32	8.43	7.62

**Table 4:** Environmental factors of the raw materials of the prepared mortar.

Raw Materials	EE (MJ/kg)	ECO_2_ (kg CO_2_/kg)	Reference
Cement	5.5	0.95	[27]
Slag	1.6	0.083	[27]
Water	0	0	[29]
Sand	0.081	0.0051	[27]
Na_2_SO_4_	4.6	0	[66]

## Data Availability

The original contributions presented in this study are included in the article. Further inquiries can be directed to the corresponding author.

## References

[1] He Z., Zhu X., Wang J., Mu M., Wang Y. (2019). Comparison of CO_2_ emissions from OPC and recycled cement production. Constr. Build. Mater..

[2] Nie S., Zhou J., Yang F., Lan M., Li J., Zhang Z., Chen Z., Xu M., Li H., Sanjayan J.G. (2022). Analysis of theoretical carbon dioxide emissions from cement production: Methodology and application. J. Clean. Prod..

[3] Skibsted J., Snellings R. (2019). Reactivity of supplementary cementitious materials (SCMs) in cement blends. Cem. Concr. Res..

[4] Jiang X., Xiao R., Bai Y., Huang B., Ma Y. (2022). Influence of waste glass powder as a supplementary cementitious material (SCM) on physical and mechanical properties of cement paste under high temperatures. J. Clean. Prod..

[5] Fu J., Jones A.M., Bligh M.W., Holt C., Keyte L.M., Moghaddam F., Foster S.J., Waite T.D. (2020). Mechanisms of enhancement in early hydration by sodium sulfate in a slag-cement blend—Insights from pore solution chemistry. Cem. Concr. Res..

[6] Wu W., Wang R., Zhu C., Meng Q. (2018). The effect of fly ash and silica fume on mechanical properties and durability of coral aggregate concrete. Constr. Build. Mater..

[7] Fu J., Bligh M.W., Shikhov I., Jones A.M., Holt C., Keyte L.M., Moghaddam F., Arns C.H., Foster S.J., Waite T.D. (2021). A microstructural investigation of a Na_2_SO_4_ activated cement-slag blend. Cem. Concr. Res..

[8] Silva L.H.P., Nehring V., De paiva F.F.G., Tamashiro J.R., Galvín A.P., López-Uceda A., Kinoshita A. (2023). Use of blast furnace slag in cementitious materials for pavements-Systematic literature review and eco-efficiency. Sustain. Chem. Pharm..

[9] Özbay E., Erdemir M., Durmuş H.İ. (2016). Utilization and efficiency of ground granulated blast furnace slag on concrete properties—A review. Constr. Build. Mater..

[10] Xiao R., Shen Z., Si R., Polaczyk P., Li Y., Zhou H., Huang B. (2022). Alkali-activated slag (AAS) and OPC-based composites containing crumb rubber aggregate: Physico-mechanical properties, durability and oxidation of rubber upon NaOH treatment. J. Clean. Prod..

[11] Güneyisi E., Gesoğlu M. (2008). A study on durability properties of high-performance concretes incorporating high replacement levels of slag. Mater. Struct..

[12] Shi C., Qian J. (2000). High performance cementing materials from industrial slags—A review, Resources. Conserv. Recycl..

[13] Ortega J., Sánchez I., Climent M. (2012). Durability related transport properties of OPC and slag cement mortars hardened under different environmental conditions. Constr. Build. Mater..

[14] Xu Z., Gao J., Zhao Y., Li S., Guo Z., Luo X., Chen G. (2022). Promoting utilization rate of ground granulated blast furnace slag (GGBS): Incorporation of nanosilica to improve the properties of blended cement containing high volume GGBS. J. Clean. Prod..

[15] Acevedo-Martinez E., Gomez-Zamorano L., Escalante-Garcia J. (2012). Portland cement-blast furnace slag mortars activated using waterglass:—Part 1: Effect of slag replacement and alkali concentration. Constr. Build. Mater..

[16] Rashad A., Bai Y., Basheer P., Milestone N., Collier N. (2013). Hydration and properties of sodium sulfate activated slag. Cem. Concr. Compos..

[17] Zhang J., Tan H., Bao M., Liu X., Luo Z., Wang P. (2021). Low carbon cementitious materials: Sodium sulfate activated ultra-fine slag/fly ash blends at ambient temperature. J. Clean. Prod..

[18] Ding Y., Shi C., Li N. (2018). Fracture properties of slag/fly ash-based geopolymer concrete cured in ambient temperature. Constr. Build. Mater..

[19] Zhu Y., Zhang Z., Yang Y., Yao Y. (2014). Measurement and correlation of ductility and compressive strength for engineered cementitious composites (ECC) produced by binary and ternary systems of binder materials: Fly ash, slag, silica fume and cement. Constr. Build. Mater..

[20] Özbay E., Karahan O., Lachemi M., Hossain K.M., Atis C.D. (2013). Dual effectiveness of freezing–thawing and sulfate attack on high-volume slag-incorporated ECC. Compos. Part B Eng..

[21] (2016). Standard Test Method for Compressive Strength of Hydraulic Cement Mortars (Using 2-in. or [50-mm] Cube Specimens). https://www.astm.org/.

[22] Xu S., Reinhardt H.W. (1999). Determination of double-K criterion for crack propagation in quasi-brittle fracture, Part I: Experimental investigation of crack propagation. Int. J. Fract..

[23] Xu S., Reinhardt H.W. (1999). Determination of double-K criterion for crack propagation in quasi-brittle fracture, Part II: Analytical evaluating and practical measuring methods for three-point bending notched beams. Int. J. Fract..

[24] Zhang B., Zhu H., Lu F. (2021). Fracture properties of slag-based alkali-activated seawater coral aggregate concrete. Theor. Appl. Fract. Mech..

[25] Wang Y., Hu S., He Z. (2021). Mechanical and fracture properties of geopolymer concrete with basalt fiber using digital image correlation. Theor. Appl. Fract. Mech..

[26] Zhang S., Li Z., Ghiassi B., Yin S., Ye G. (2021). Fracture properties and microstructure formation of hardened alkali-activated slag/fly ash pastes. Cem. Concr. Res..

[27] Long W., Wu Z., Khayat K.H., Wei J., Dong B., Xing F., Zhang J. (2022). Design, dynamic performance and ecological efficiency of fiber-reinforced mortars with different binder systems: Ordinary Portland cement, limestone calcined clay cement and alkali-activated slag. J. Clean. Prod..

[28] Li Y., Zeng X., Zhou J., Shi Y., Umar H.A., Long G., Xie Y. (2021). Development of an eco-friendly ultra-high performance concrete based on waste basalt powder for Sichuan-Tibet Railway. J. Clean. Prod..

[29] Xiao R., Ma Y., Jiang X., Zhang M., Zhang Y., Wang Y., Huang B., He Q. (2020). Strength, microstructure, efflorescence behavior and environmental impacts of waste glass geopolymers cured at ambient temperature. J. Clean. Prod..

[30] Rahman M.A., Sarker P.K., Shaikh F.U.A., Saha A.K. (2017). Soundness and compressive strength of Portland cement blended with ground granulated ferronickel slag. Constr. Build. Mater..

[31] Yazıcı H., Yardımcı M.Y., Yiğiter H., Aydın S., Türkel S. (2010). Mechanical properties of reactive powder concrete containing high volumes of ground granulated blast furnace slag. Cem. Concr. Compos..

[32] Dos Santos V.H.J.M., Pontin D., Ponzi G.G.D., E Stepanha A.S.D.G., Martel R.B., Schütz M.K., Einloft S.M.O., Vecchia F.D. (2021). Application of Fourier Transform infrared spectroscopy (FTIR) coupled with multivariate regression for calcium carbonate (CaCO3) quantification in cement. Constr. Build. Mater..

[33] Lee H.K., Jeon S., Lee B.Y., Kim H. (2020). Use of circulating fluidized bed combustion bottom ash as a secondary activator in high-volume slag cement. Constr. Build. Mater..

[34] Abdulkareem O.M., Fraj A.B., Bouasker M., Khouchaf L., Khelidj A. (2021). Microstructural investigation of slag-blended UHPC: The effects of slag content and chemical/thermal activation. Constr. Build. Mater..

[35] Cai G., Zhou Y., Li J., Han L., Poon C.S. (2022). Deep insight into mechanical behavior and microstructure mechanism of quicklime-activated ground granulated blast-furnace slag pastes. Cem. Concr. Compos..

[36] Deng G., He Y., Lu L., Hu S. (2020). The effect of activators on the dissolution characteristics and occurrence state of aluminum of alkali-activated metakaolin. Constr. Build. Mater..

[37] Gao X., Yao X., Wang C., Geng C., Yang T. (2022). Properties and microstructure of eco-friendly alkali-activated slag cements under hydrothermal conditions relevant to well cementing applications. Constr. Build. Mater..

[38] Zhang J., Tan H., He X., Yang W., Deng X. (2020). Utilization of carbide slag-granulated blast furnace slag system by wet grinding as low carbon cementitious materials. Constr. Build. Mater..

[39] Dixit A., Geng G., Du H., Pang S.D. (2022). The role of age on carbon sequestration and strength development in blended cement mixes. Cem. Concr. Compos..

[40] Wu M., Zhang Y., Jia Y., She W., Liu G., Yang Y., Rong Z., Sun W. (2019). The influence of chemical admixtures on the strength and hydration behavior of lime-based composite cementitious materials. Cem. Concr. Compos..

[41] Ding Y., Dai J., Shi C. (2018). Fracture properties of alkali-activated slag and ordinary Portland cement concrete and mortar. Constr. Build. Mater..

[42] Königsberger M., Carette J. (2020). Validated hydration model for slag-blended cement based on calorimetry measurements. Cem. Concr. Res..

[43] Hallet V., Pedersen M.T., Lothenbach B., Winnefeld F., De Belie N., Pontikes Y. (2022). Hydration of blended cement with high volume iron-rich slag from non-ferrous metallurgy. Cem. Concr. Res..

[44] Yang K., White C.E. (2021). Modeling of aqueous species interaction energies prior to nucleation in cement-based gel systems. Cem. Concr. Res..

[45] Lodeiro I.G., Macphee D., Palomo A., Fernández-jiménez A. (2009). Effect of alkalis on fresh C–S–H gels. FTIR analysis. Cem. Concr. Res..

[46] Kapeluszna E., Kotwica Ł., Różycka A., Gołek Ł. (2017). Incorporation of Al in C-A-S-H gels with various Ca/Si and Al/Si ratio: Microstructural and structural characteristics with DTA/TG, XRD, FTIR and TEM analysis. Constr. Build. Mater..

[47] Li H., Yang K., Guan X. (2019). Properties of sulfoaluminate cement-based grouting materials modified with LiAl-layered double hydroxides in the presence of PCE superplasticizer. Constr. Build. Mater..

[48] Trezza M., Rahhal V.F. (2019). Self-activation of slag-cements with glass waste powder. Mater. Construcción.

[49] Chen Z., Lee Y., Cho H., Lee H., Lim S. (2019). Improvement in carbonation resistance of portland cement mortar incorporating γ-dicalcium silicate. Adv. Mater. Sci. Eng..

[50] Taddei P., Tinti A., Gandolfi M.G., Rossi P., Prati C. (2009). Vibrational study on the bioactivity of Portland cement-based materials for endodontic use. J. Mol. Struct..

[51] Ahmad M.R., Qian L., Fang Y., Wang A., Dai J. (2023). A multiscale study on gel composition of hybrid alkali-activated materials partially utilizing air pollution control residue as an activator. Cem. Concr. Compos..

[52] Lothenbach B., Jansen D., Yan Y., Schreiner J. (2022). Solubility and characterization of synthesized 11 Å Al-tobermorite. Cem. Concr. Res..

[53] Guo X., Meng F., Shi H. (2017). Microstructure and characterization of hydrothermal synthesis of Al-substituted tobermorite. Constr. Build. Mater..

[54] Bernard E., Yan Y., Lothenbach B. (2021). Effective cation exchange capacity of calcium silicate hydrates (C-S-H). Cem. Concr. Res..

[55] Garg N., Özçelik V.O., Skibsted J., White C.E. (2019). Nanoscale Ordering and Depolymerization of Calcium Silicate Hydrates in the Presence of Alkalis. J. Phys. Chem. C.

[56] Li Y., Yin J., Yuan Q., Huang L., Li J. (2022). Greener strain-hardening cementitious composites (SHCC) with a novel alkali-activated cement. Cem. Concr. Compos..

[57] Li N., Farzadnia N., Shi C. (2017). Microstructural changes in alkali-activated slag mortars induced by accelerated carbonation. Cem. Concr. Res..

[58] Zhang J., Shi C., Zhang Z. (2019). Carbonation induced phase evolution in alkali-activated slag/fly ash cements: The effect of silicate modulus of activators. Constr. Build. Mater..

[59] Plevová E., Vaculikova L., Kozusnikova A., Ritz M., Simha Martynková G. (2016). Thermal expansion behaviour of granites. J. Therm. Anal. Calorim..

[60] Zhang J., Shi C., Zhang Z. (2021). Effect of Na_2_O concentration and water/binder ratio on carbonation of alkali-activated slag/fly ash cements. Constr. Build. Mater..

[61] Senvaitiene J., Smirnova J., Beganskiene A., Kareiva A. (2007). XRD and FTIR Characterisation of Lead Oxide-Based Pigments and Glazes. Acta Chim. Slov..

[62] Schmidt-döhl F.M., Schulenberg D., Tralow F., Neubauer J., Wolf J.J., Ectors D. (2022). Quantitative analysis of the strength generating C-S-H-phase in concrete by IR-spectroscopy. Acta Polytech. CTU Proc..

[63] Chen W., Li B., Wang J., Thom N. (2021). Effects of alkali dosage and silicate modulus on autogenous shrinkage of alkali-activated slag cement paste. Cem. Concr. Res..

[64] Zhang G., Li Y., Yang J., Ding Q., Sun D. (2021). Insight into the strengthening mechanism of the al-induced cross-linked calcium aluminosilicate hydrate gel: A molecular dynamics study. Front. Mater..

[65] Wu M., Zhang Y., Jia Y., She W., Liu G., Yang Z., Zhang Y., Zhang W., Sun W. (2019). Effects of sodium sulfate on the hydration and properties of lime-based low carbon cementitious materials. J. Clean. Prod..

[66] CKulasuriya, Vimonsatit V., Dias W. (2021). Performance based energy, ecological and financial costs of a sustainable alternative cement. J. Clean. Prod..

[67] Johari M.M., Brooks J., Kabir S., Rivard P. (2011). Influence of supplementary cementitious materials on engineering properties of high strength concrete. Constr. Build. Mater..

[68] Mota B., Matschei T., Scrivener K. (2018). Impact of NaOH and Na_2_SO_4_ on the kinetics and microstructural development of white cement hydration. Cem. Concr. Res..

[69] Hou D., Wu C., Yang Q., Zhang W., Lu Z., Wang P., Li J., Ding Q. (2020). Insights on the molecular structure evolution for tricalcium silicate and slag composite: From 29Si and 27Al NMR to molecular dynamics. Compos. Part B Eng..

[70] García lodeiro I., Fernández-jimenez A., Palomo A., Macphee D. (2010). Effect on fresh C-S-H gels of the simultaneous addition of alkali and aluminium. Cem. Concr. Res..

[71] Wei S., Zheng K., Chen W., Chen L., Zhou J., Mi T. (2023). The correlation between Al incorporation and alkali fixation by calcium aluminosilicate hydrate: Observations from hydrated C3S blended with and without metakaolin. Cem. Concr. Res..

[72] Fan D., Yang S. (2018). Mechanical properties of C-S-H globules and interfaces by molecular dynamics simulation. Constr. Build. Mater..

[73] Yaphary Y.L., Lau D., Sanchez F., Poon C.S. (2020). Effects of sodium/calcium cation exchange on the mechanical properties of calcium silicate hydrate (C-S-H). Constr. Build. Mater..

[74] CPlassard, Lesniewska E., Pochard I., Nonat A. (2005). Nanoscale experimental investigation of particle interactions at the origin of the cohesion of cement. Langmuir.

